# The Emulsion Properties of Chicken Liver Protein Recovered through Isoelectric Solubilization/Precipitation Processes

**DOI:** 10.3390/foods11111644

**Published:** 2022-06-02

**Authors:** Shunchang Pu, Cong Yin, Xingguo Zhang, Yu Zhang, Ning Lu, Guoyuan Xiong

**Affiliations:** 1 Bozhou Key Laboratory of Medicinal and Edible Homology Functional Food, Department of Biology and Food Engineering, Bozhou University, Bozhou 236800, China; pcc922800@163.com (S.P.); cauzhangyu@163.com (Y.Z.); mhxy826701264@163.com (N.L.); 2 Anhui Engineering Laboratory for Agro-Products Processing, College of Tea & Food Science and Technology, Anhui Agricultural University, Hefei 230036, China; 2353806763@stu.ahau.edu.cn (C.Y.); xingguoz@stu.ahau.edu.cn (X.Z.)

**Keywords:** chicken liver protein, isoelectric solubilization/precipitation, emulsifying properties, protein recovery

## Abstract

This study investigated the feasibility to improve the emulsifying capacity of chicken liver (CL) protein using different isoelectric solubilization/precipitation (ISP) processes. The CL proteins were first solubilized at alkaline pH 10.5, 11.0, 11.5, and 12.0, followed by precipitation at pH 5.0, 5.5, and 6.0, respectively. Fresh CL paste was set as the control (raw). With the increase in solubilization pH, the protein recovery yield increased under the same precipitation pH, and the pH 12.0, 5.5 treatment obtained the highest recovery yield of 82% (*p* < 0.05), followed by the pH 5.0 precipitation treatments and the pH 12.0, 6.0 treatment. The particle size distribution of D_3,2_ and D_4,3_ was smaller for the pH 10.5 (except for the D_4,3_ of pH 10.5, 5.0) and pH 11.0 solubilization treatments than those of the other treatments (*p* < 0.05), regardless of precipitation pH. Compared with that of the raw control, the emulsions of the pH 10.5 and pH 11.0 solubilization treatments, and pH 12.0, 6.0 treatment showed good stability. The pH 10.5, 6.0 treatment showed the best emulsification activity, followed by the pH 10.5, 5.5, pH 11.0, 6.0, pH 12.0, 6.0, pH 10.5, 5.0, pH 11.0, 5.5, and pH 11.0, 5.0 treatments, which were uniformly distributed and were stable without the stratification of emulsions. It was concluded that CL protein recovered through suitable ISP showed potential as an emulsifier, and thus expanded the application of CL protein for human consumption.

## 1. Introduction

Chicken liver (CL) in physiology is not only a major contributor to protein production with many types of protein compositions, but also contains up to 20% high quality protein (with a well-balanced essential amino acid composition), which is equivalent to the protein content in livestock and poultry meat [1,2,3]. However, its poor emulsification capacity (EC), unpleasant flavor, taste, and high cholesterol content reduce its acceptance amongst consumers and make it a de-valued animal co-product [4]. At present, most CL is utilized for animal feed with a low economic value, and part of it is used to cook, braise, marinate, or produce chicken liver paste in China [5]. The strategy to ameliorate the economic value of CL is fascinating for food producers and the chicken industry. As an important chemical component, the protein plays a crucial role in the quality of livestock and poultry products, directly affecting the taste, texture, and other sensory qualities of its products. Taking into account the high protein content and valuable source of CL, it is feasible to isolate its high-quality protein for further utilization [4].

The current extraction methods for the protein of co-products include enzyme-assisted extraction, cavitation-assisted extraction, aqueous extraction, and organic solvent, etc. [6]. Nonetheless, more coherent and holistic studies are needed to establish sustainable extraction technologies for the protein from animal co-products to produce high-quality protein products and to meet the needs of human consumption. ISP stands for isoelectric solubilization/precipitation, which is a method for inducing the solubilization of protein in co-products under extreme alkaline or acidic pH, and then precipitation through adjusting the pH to the isoelectric point (pI) to separate and extract protein, based on the theory that muscle proteins have a different solubility under extreme acid or base conditions, also known as acid–base processing or pH shift [7]. Nolsøe and Undeland [8] reported that ISP has been applied in the food industry to separate fish protein and for the mechanical separation of protein and to improve their functional properties. Over the past few years, ISP has been extensively considered as an efficient technology to modify the functional properties of protein [9]. It is recognized as an eco-friendly and low-cost technique for industrial application in order to extract protein and improve the emulsifying properties in fish [10], muscle-based products [11], and goose liver [12]. During the ISP process, proteins undergo partial unfolding and then refolding because of the extreme acid or base conditions, leading to changes in the protein conformation and structure, and thus affecting the processing properties of the protein, especially the emulsifying properties. The emulsifying properties of proteins are key for the applications of many foods, and are generally expressed in the form of emulsifying activity (EA) and emulsifying stability (ES). Recently, some studies have shown that the EA and ES of the proteins obtained from different raw materials through different ISPs are different [9,12,13]. Although it has been reported that ISP causes physicochemical property changes to CL protein and might enhance its ES [7], the relationship between solubilization/precipitation pH and the emulsifying properties is far from understood.

Therefore, in this study, to elucidate such aspects, the CL protein was subjected to alkaline solubilization (pH 10.5, pH 11.0, pH 11.5, and pH 12.0) and then precipitation at pH 5.0, pH 5.5, and pH 6.0, respectively, to examine the effect of solubilization/precipitation pH on the emulsifying properties through analyzing the EA, ES, microphotographs, and visual appearance of their emulsions. It is of great practical importance to actively develop innovative technologies of protein processing, such as the ISP method, in order to fully utilize the de-valued co-products and to improve the emulsifying characteristics of their proteins for industrial food applications.

## 2. Materials and Methods

### 2.1. Materials

About 15kg fresh CL was purchased from local commercial slaughterhouses (Liu Laoer Roast Chicken Co., LTD, Suzhou, China). After being trimmed to remove the bile ducts and larger blood vessels, the CL was ground using a grinder (MM-12, 8 mm plate, Guangdong, China) and was mixed thoroughly to obtain fresh CL batters. Then, it was packed into polyethylene bags (~500 g), sealed, and frozen (−20 °C) until use within 15 days. The day before the preparation of the experiment, the frozen CL batters were thawed overnight in a refrigerator at 4℃. In this study, all of the chemicals and reagents used were of analytical grade.

### 2.2. Methods

#### 2.2.1. CL Protein Extraction

CL protein extraction was conducted as described by Xiong, Gao, Wang, Xu, and Zhou [7], in detail. Briefly, the diluted slurry of homogenized CL was subjected to the alkaline (pH 10.5, 11.0, 11.5, and 12.0) for 10 min at 4 °C to solubilize the proteins via electrostatic repulsion. Then, the insoluble materials, such as cellular membranes, connective tissue, and lipids were separated through centrifugation. The soluble CL protein was adjusted to a final pH of 5.0, 5.5, and 6.0, for 10 min, respectively, to be precipitated and were finally collected through centrifugation at 10,000× *g* for 10 min at 4 °C to obtain the ISP CL protein extractions.

#### 2.2.2. Recovery Yield

The recovery yield of CL protein was determined according to Xiong, Gao, Wang, Xu, and Zhou [7], with slight modification. The total protein total amount of fresh CL and ISP CL protein extractions obtained from the procedure presented in Section 2.2.1 were examined separately through the Kjeldahl method using a FOSS 2300 Automatic Kjeldahl Nitrogen Determination Analyzer (Switzerland FOSS Company, Hillerod, Denmark). The recovery yield was calculated using the ratio of the total amount of isolated protein to the total protein content of fresh CL.

#### 2.2.3. Particle Size and Distribution

The particle size and size distribution of the CL proteins after different ISP treatments were monitored separately using a MS-2000 Dynamic Light Scattering Particle Sizer (Malvern Instrument, Worcestershire, UK). Deionized water was used as the dispersant, and the parameters were set to a refractive index of 1.47 for the samples and 1.330 for the dispersant. CL proteins from different ISPs were slowly added to the dynamic light scattering particle size meter for testing. The volume fraction D_4,3_, specific surface fraction D_3,2_, and particle size distribution of the proteins were detected.

#### 2.2.4. Preparation of Emulsions

Before the emulsion preparation, the content of fresh CL protein and different ISP CL protein extractions was determined using the Kjeldahl method. To prepare the emulsions, 21 g of soybean oil and 9 g of distilled water were added to about 50 g CL protein extractions or CL (after measuring their protein content to ensure there was 1.8 g protein in every sample). Then, the mixtures were homogenized using an IKA T25 homogenizer at 10,000 rpm twice for 30 s and at 15,000 rpm thrice for 30 s in an ice bath. Finally, the final emulsions were stored at 4 °C for further evaluation.

#### 2.2.5. Emulsion Activity

The emulsion activity index (EAI) was measured following the method of Hrynets, et al. [14]. Briefly, after homogenization, 0.05 mL of the freshly prepared emulsion was immediately diluted to 5 mL with 1 g/L of sodium dodecyl sulfate (SDS) solution. The absorbance was measured at 500 nm using a spectrophotometer (PE-Lambda, Waltham, Massachusetts, USA) at 0 min *(A*_0_). The EAI was calculated based on the following equation: EAI = 2.33 × *A*_0_.

#### 2.2.6. Emulsion Stability

Freshly prepared emulsions (added NaN_3_, 0.4 g/L) were each transferred into 10 mL glass bottles and were stored at 20 °C room temperature for visual inspection of the height differences of the emulsion layers during storage for 8 d. Photographs were taken at 4 d and 8 d.

#### 2.2.7. Optical Microscopy Observation

Freshly prepared emulsions (50 µL) were separately dropped on a microscope slide and were quickly covered with a coverslip. These emulsions were observed at ambient temperature using optical microscopy (Olympus, 1X51, Shanghai, China), as previously described by Zhao et al. [15]. Microphotographs of the emulsions were taken using a 40× objective lens.

#### 2.2.8. Statistical Analysis

All of the experiments were independently conducted at least three times on different occasions. Each test was performed at least three times (n = 3). The data were expressed as means ± standard deviation (SD). Analysis of variance (ANOVA) was implemented using SPSS 22.0 (SPSS Inc., Chicago, Illinois, USA). Differences between mean values were decided with t-test at *p* < 0.05. The charts were drawn using Origin 2019 pro software.

## 3. Results and Discussion

### 3.1. Recovery Yield

Protein recovery yield is a key parameter for determining the feasibility and economy of an extraction technique. Protein production during acid or base treatments is determined by the following three main factors: protein solubility under extreme acidic or basic conditions, the amount of precipitate formed during centrifugation, and protein precipitation at isoelectric point pH [8].

**Figure 1:**
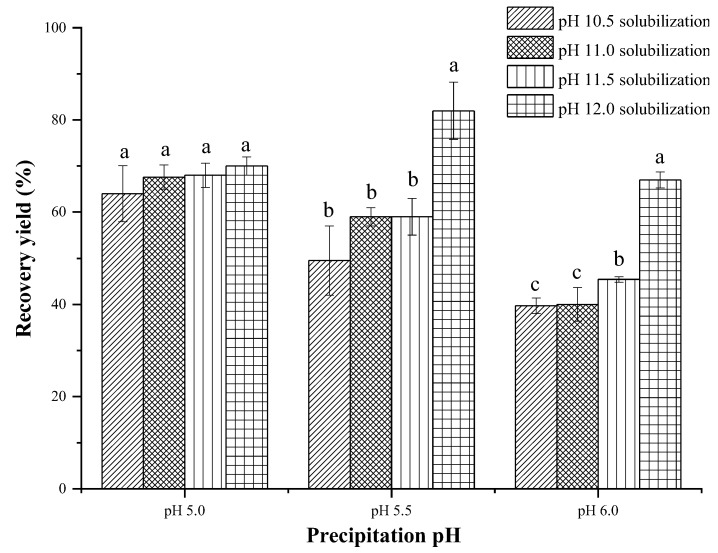
Recovery yield of chicken liver protein isolated using different ISPs. Different letters (a–c) indicate significant differences within the same precipitation pH treatments (*p* < 0.05).

It is evident from Figure 1 that CL protein recovery yield had an increasing tendency with the increase in solubilization pH under the same precipitation pH. However, no significant difference was detected for the pH 5.0 precipitation treatments, even for solubilization with different pH (*p* > 0.05). When precipitation with pH 5.0, the recovery yields of the CL protein at pH 10.5, 11.0, and 11.5 solubilization were correspondingly higher than for the pH 5.5 and 6.0 precipitation treatments (*p* < 0.05). Interestingly, the pH 5.5 precipitation treatment obtained the highest CL protein recovery yield (*p* < 0.05) of 82% with pH 12.0 solubilization. Xiong, Gao, Wang, Xu, and Zhou [7] found that the highest protein recovery yield was significantly associated with the solubility profile of the CL protein, and showed the highest solubility at pH 12.0 and the highest insolubility at pH 5.0 and 5.5. For all of the treatments, a lower CL protein recovery yield was found when precipitated with pH 6.0 treatment, regardless of the solubilization pH treatment, and the lowest recovery found was of 39.7% in the pH 10.5, 6.0 treatment, which could be related to the far protein isoelectric point. Kristinsson [16] found that the protein recovery yields of tilapia were 56 to 61% for the acid solubilization and 61 to 68% for the alkali solubilization. The study of Abdollahi and Undeland [17] also showed that a pH higher than 11.0 enhanced the protein solubility and improved the recovery yield of fish proteins. The higher solubility of molecules in alkaline solutions might explain the high protein recovery [7]. The present experiment was similar to the above results, that an extreme alkaline pH produced higher protein recovery. A sufficiently high pH was a prerequisite to obtain CL homogenates for the separation of fascial fat during the first step of centrifugation, and maximized the solubility of CL proteins through max degree denaturation of the proteins at these conditions [17]. Appropriate isoelectric point precipitation was also essential for protein collection [7].

### 3.2. Particle Size and Distribution

**Figure 2:**
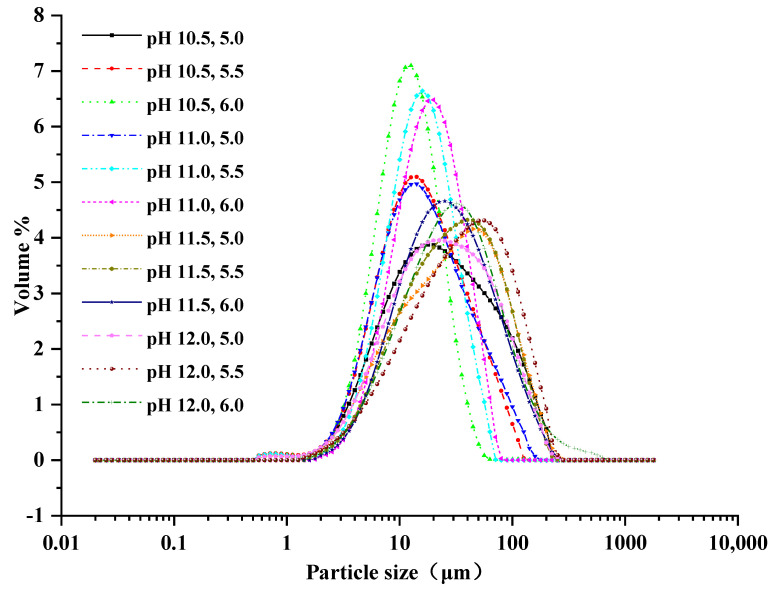
The distributions of the particle size of chicken liver protein recovered using different ISPs. pH 10.5, 5.0: pH 10.5 solubilization and pH 5.0 precipitation; pH 10.5, 5.5: pH 10.5 solubilization and pH 5.5 precipitation; pH 10.5, 6.0: pH 10.5 solubilization and pH 6.0 precipitation; pH 11.0, 5.0: pH 11.0 solubilization and pH 5.0 precipitation; pH 11.0, 5.5: pH 11.0 solubilization and pH 5.5 precipitation; pH 11.0, 6.0: pH 11.0 solubilization and pH 6.0 precipitation; pH 11.5, 5.0: pH 11.5 solubilization and pH 5.0 precipitation; pH 11.5, 5.5: pH 11.5 solubilization and pH 5.5 precipitation; pH 11.5, 6.0: pH 11.5 solubilization and pH 6.0 precipitation; pH 12.0, 5.0: pH 12.0 solubilization and pH 5.0 precipitation; pH 12.0, 5.5: pH 12.0 solubilization and pH 5.5 precipitation; pH 12.0, 6.0: pH 12.0 solubilization and pH 6.0 precipitation.

The particle size of the protein is one of the important factors for a desirable emulsifying capacity of protein [11]. To evaluate the changes in protein particle size after using ISP treatments, the particle size distribution and D_3,2_ and D_4,3_ values of the CL protein precipitated with different pH in each of the solubilization treatments are presented in Figure 2 and Table 1, respectively.

The particle size distribution of the ISP treatments all exhibited one primary peak. However, different particle size distributions were observed with different ISP treatments, and the curves of the pH 10.5, 6.0, pH 11.0, 5.5, pH 11.0, 6.0, pH 10.5, 5.5, and pH 11.0, 5.0 treatments were higher and had a narrower peak, and moved relatively to the left compared with those of the other ISP treatments. In particular, the curve of the pH 10.5, 6.0 treatment moved to the far left, which indicated that the particle sizes were mainly concentrated in the smallest region [9], as mentioned in Table 1. Conversely, it presented the biggest particle size for other ISP treatments; in particular, the curve of the pH 12.0, 5.5 moved to the far right, which had the largest D_3,2_ and D_4,3_ values, as shown in Table 1.

The D_3,2_ value, which is the particle size to surface area, decreased in the solubilization pH 10.5 treatments and increased in the solubilization pH 11.0 treatments as the precipitation pH gradually increased. Nevertheless, the D_3,2_ values first increased and then decreased as the precipitation pH gradually increased in the solubilization pH 11.5 and 12.0 treatments. As for the D_4,3_ value, which was particle size to volume, it gradually decreased in the solubilization pH 10.5 and 11.5 treatments, but first decreased and then increased in the solubilization pH 11.0 treatments, and first increased and then decreased in the solubilization pH 12.0 treatments as the precipitation pH increased. As mentioned above, the smallest and largest values of D_3,2_ and D_4,3_ were found for the pH 10.5, 6.0 and pH 12.0, 5.5 treatments, respectively (*p* < 0.05). These results suggest that the different ISP treatments could increase or reduce the aggregation of the protein, resulting in bigger or smaller particle size and with a different emulsifying capacity. Furthermore, the D_4,3_ values were larger than the D_3,2_ values in all of the treatments (Table 1), indicating that there were more larger particles than smaller particles [18]. Alkaline pH solubilization favored the collection of larger particle sizes for CL proteins, which partially unfolded when the muscle proteins were exposed to extreme pH, and this breakdown caused significant changes in the protein conformation and structure [19]. The stability of emulsions was inextricably linked to the particle size of the protein [20]. Differences in the particle size of ISP-extracted CL protein might cause differences in processing functionality in practical applications [21].

### 3.3. Emulsion Activity Index (EAI)

The emulsion activity index (EAI) refers to the area of interfacial membrane stabilized per unit weight of protein (cm^2^/mg), indicating the ability of proteins to adsorb on the oil–water interface during emulsification. EAI is also one of the important properties for the emulsification capacity (EC), which can reflect the potentials of the proteins for application in a variety of emulsion-based food products [22].

**Figure 3:**
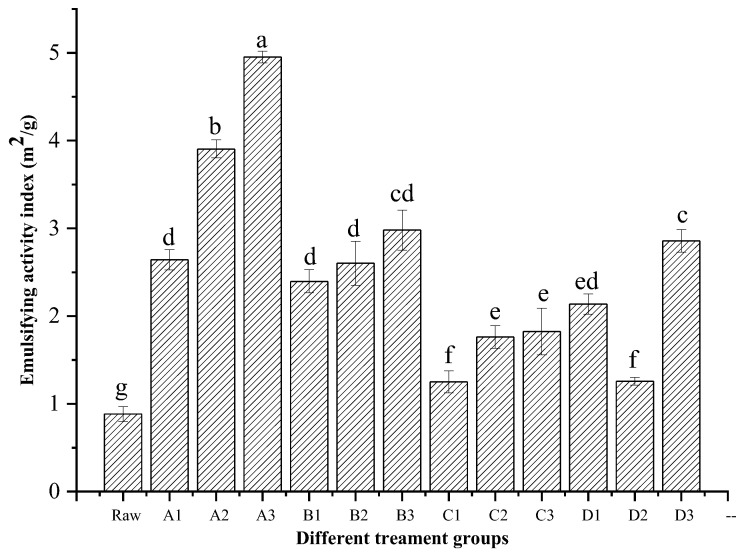
Emulsion activity index (EAI) of chicken liver protein recovered by different ISPs. Different letters (a–g) indicate significant differences (*p* < 0.05). Raw: raw chicken liver; A1–A3: pH 10.5 solubilization, and pH 5.0, pH 5.5 and pH 6.0 precipitation, respectively; B1–B3: pH 11.0 solubilization, and pH 5.0, pH 5.5 and pH 6.0 precipitation, respectively; C1–C3: pH 11.5 solubilization, and pH 5.0, pH 5.5 and pH 6.0 precipitation, respectively; D1–D3: pH 12.0 solubilization, and pH 5.0, pH 5.5 and pH 6.0 precipitation, respectively.

The EAI of raw chicken liver and CL protein recovered by different ISP are shown in Figure 3. The raw chicken liver showed a minimum EAI (*p* < 0.05). Interestingly, after being processed by ISP, the EAI of the proteins were all increased (*p* < 0.05), which might be related to their better emulsification capacity. Xue, Yu, Li, Zhao, Han, Xu, and Zhou [12] also reported that the EC of goose liver protein was improved by alkaline processes (pH 11.0, 11.5, and 12.0 solubilization, and pH 5.5 precipitation). The high EAI of CL protein recovered by ISP might be related to the partial unfolding and denaturation of proteins experienced during the ISP process, which provided rapid adsorption of the relatively hydrophobic globular head of the pH-treated protein to the nonpolar lipid globules [23]. Kristinsson [24] pointed out that there was a very good relationship between the increase in surface hydrophobicity and the EAI of ISP-treated cod myosin. This behavior could also be explained because there was greater favoring of the hydrophilic region of the ISP CL protein, which increased the EC [25]. Under the same precipitation pH condition, CL proteins at solubilization pH 10.5 and 11.0 treatments presented higher EAI values, which had a relatively moderate extreme pH compared with those at extreme solubilization pH 11.5 and 12.0 treatments (except for the pH 12.0, 6.0 treatment). The higher EAI of CL protein could be related to its higher surface hydrophobicity and favorable EC [22]. Therefore, we deduced that CL protein with a better emulsifying performance might be obtained through moderate extreme pH solubilization. The maximum EAI of the recovered CL protein was obtained for the pH 10.5, 6.0 treatment, followed by the pH 10.5, 5.5, pH 12.0, 6.0, and pH 11.0, 6.0 treatments. The process of conformational changes at the oil–water interface was due to a loss in tertiary structure rather than secondary structure during protein emulsification. We hypothesize that the unfolding level of the tertiary structure of CL protein would be different with the increase in extreme solubilization pH. When CL protein was precipitated using a different near isoelectric point pH, a difference in refolding level was again produced, which resulted in a different EAI. Furthermore, the increasing EAI could also be explained by the reduction in the particle size of the protein, as above mentioned in Table 1 and Figure 2. Zou et al. [26] observed that a smaller particle size increased the EAI of the protein. Hence, appropriate ISP treatment should be an effective method to improve the emulsifying properties of CL protein.

### 3.4. Emulsion Stability Index (ESI)

ESI is the ability of proteins to oppose the phase separation of the emulsion turbidity. The ability of muscle proteins to emulsify fat is of much importance in minced meat products [24]. Generally, the ES can be directly observed through the stratification of the visual appearance of emulsions by over different days to assess the quality of the emulsion.

**Figure 4:**
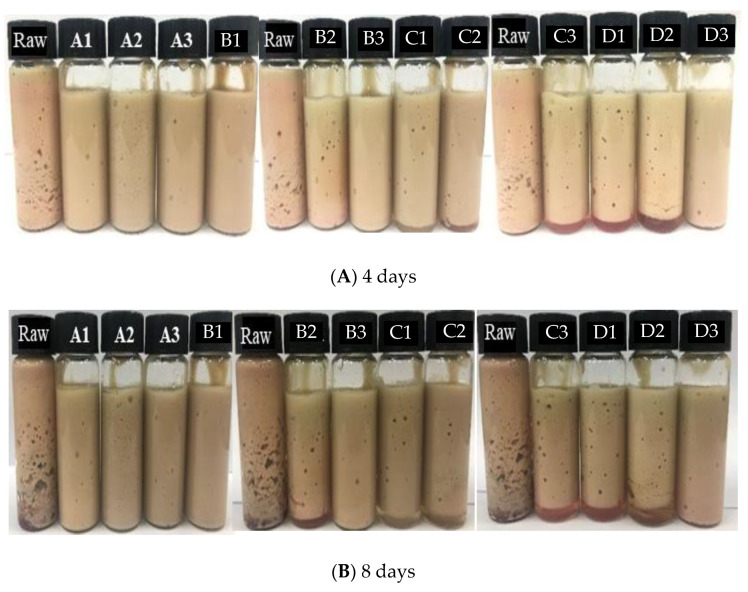
Visual appearance of emulsions prepared with raw chicken liver and chicken liver proteins recovered by different ISP when aged for 4 d (**A**) and 8 d (**B**) respectively. Raw: raw chicken liver; A1–A3: pH 10.5 solubilization, and pH 5.0, pH 5.5 and pH 6.0 precipitation, respectively; B1–B3: pH 11.0 solubilization, and pH 5.0, pH 5.5 and pH 6.0 precipitation, respectively; C1–C3: pH 11.5 solubilization, and pH 5.0, pH 5.5 and pH 6.0 precipitation, respectively; D1–D3: pH 12.0 solubilization, and pH 5.0, pH 5.5 and pH 6.0 precipitation, respectively.

The emulsification properties of raw CL and ISP CL protein were measured by its ability to emulsify and stabilize soybean oil in water. No stratification was found for the emulsion of raw CL and ISP CL protein after being placed for the first day, and the emulsion tended to be stable. However, impairment was found in the emulsion of raw CL, which appeared to have some small holes, and a loose and rough appearance, after being placed for 4 days (Figure 4A). Although the emulsions of ISP CL proteins all presented a fine visual appearance and better ES than that of raw CL after being placed for 4 days, as the solubilization pH condition used grew more extreme (pH 11.5 and pH 12.0 solubilization treatments, except for pH 12.0, 6.0 treatment), the EC of the CL proteins grew worse and stratification occurred. The results were in good agreement with the findings of Zhao et al. [27], who found that the ES of PSE-like chicken breast protein extracted at more extreme pH became worse. Xue, Yu, Li, Zhao, Han, Xu, and Zhou [12] also found that the ES of alkaline pH 11.0 solubilization goose liver proteins was better than those of extreme acid solubilization (pH 2.0, 2.5 and 3.0) or extreme alkaline solubilization (pH 11.5 and 12.0) goose liver proteins. As for the pH 12.0, 6.0 treatment, this might explain why it had a better ESI, because a moderate exposure of protein (although solubilized using extreme pH, refolded using relatively neutral pH 6.0) facilitated the enhancement of the emulsifying stability [28].

**Figure 5:**
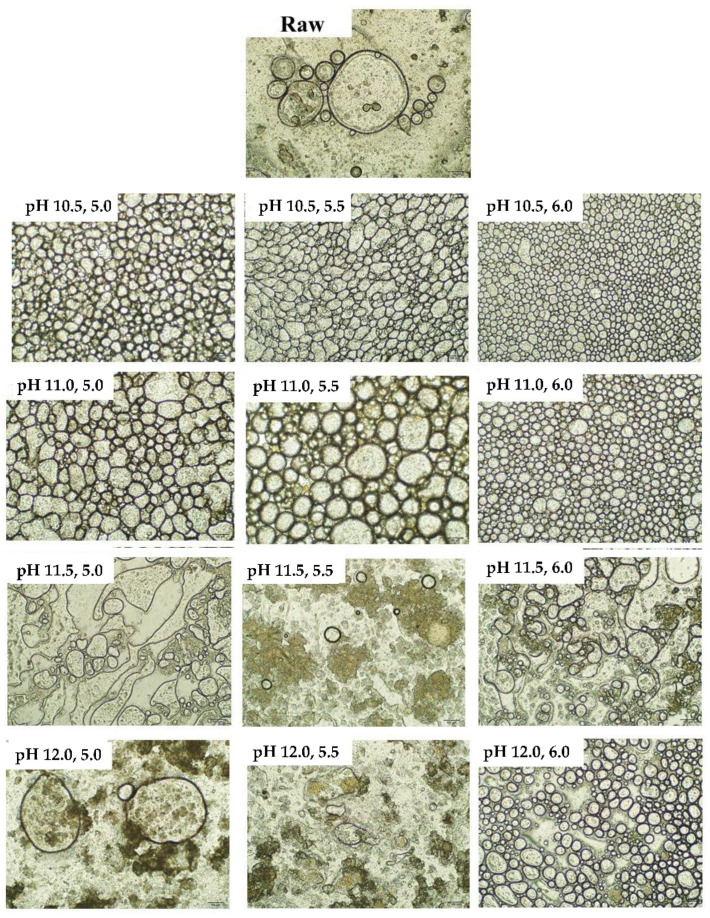
Microphotographs for the emulsions prepared with raw chicken liver and chicken liver proteins recovered using different ISP (the bar length represents 100 μm). Raw: raw chicken liver; pH 10.5, 5.0: pH 10.5 solubilization and pH 5.0 precipitation; pH 10.5, 5.5: pH 10.5 solubilization and pH 5.5 precipitation; pH 10.5, 6.0: pH 10.5 solubilization and pH 6.0 precipitation; pH 11.0, 5.0: pH 11.0 solubilization and pH 5.0 precipitation; pH 11.0, 5.5: pH 11.0 solubilization and pH 5.5 precipitation; pH 11.0, 6.0: pH 11.0 solubilization and pH 6.0 precipitation; pH 11.5, 5.0: pH 11.5 solubilization and pH 5.0 precipitation; pH 11.5, 5.5: pH 11.5 solubilization and pH 5.5 precipitation; pH 11.5, 6.0: pH 11.5 solubilization and pH 6.0 precipitation; pH 12.0, 5.0: pH 12.0 solubilization and pH 5.0 precipitation; pH 12.0, 5.5: pH 12.0 solubilization and pH 5.5 precipitation; pH 12.0, 6.0: pH 12.0 solubilization and pH 6.0 precipitation.

As displayed in Figure 4B, although the stratification of raw CL, pH 11.5, 5.0, pH 11.5, 5.5, pH 11.5, 6.0, pH 12.0, 5.0, and pH 12.0, 5.5 treatments was intensified, and the pH 11.0, 5.5 treatment was also stratified when placed for 8 days, the ES of other ISP treatments was still well maintained. The ability of the protein to stabilize the emulsion was related to the interface area of the coated protein, that is, the particle size of protein [29], the uniformity of the emulsion droplet distribution in the emulsion [30], as mentioned below in Figure 5, and the moderate exposure of protein hydrophobic groups [12]. As mentioned above, the solubilization pH 10.5 and 11.0 treatments all had a relatively small particle size distribution, which might have facilitated the formation of a stable interface film layer and improved the ESI compared with other ISP proteins. Meanwhile, this tendency also generally coincided with the tendency that was observed in the EAI of the CL protein (Figure 3).

### 3.5. Microscopic Observation of Emulsion

The inverted microscopic images of the emulsions of the raw CL and CL protein recovered with different ISPs are recorded in Figure 5. The optical inverted microscope clearly reflects the droplet distribution of the emulsion, and it can be clearly seen that the droplets of the raw CL emulsion were larger and inconsistent in size with an extremely uneven distribution. Compared with the raw emulsion, the CL protein emulsions with different ISP treatments showed different and good droplet distributions, which were related to the adsorption ability of the CL protein at the oil–water interface. This might be because different ISP treatments boosted stronger intramolecular electrostatic repulsions, which resulted in more extensive unfolding and a higher protein hydrophobicity [31], and protein hydrophobic groups can adsorb strongly to the oil–water interface to improve the emulsifying properties of the ISP CL protein. Meanwhile, disorganized the protein adsorption of a relatively smaller particle size might result in steric hindrance to stabilize the emulsion [9].

The droplet distributions of the pH 10.5 and pH 11.0 solubilization treatments were all significantly smaller, regular in shape, and homogeneous in the continuous phase. Nevertheless, extreme pH 11.5 and pH 12.0 solubilization treatments were observed to have relatively large and irregular oil droplets. Interestingly, the pH 12.0, 6.0 treatment was also observed a uniform and dispersed oil droplet distribution. The differences in the morphology of the microscopic images were in general agreement with the above results for the particle size distribution and emulsifying capacity.

## 4. Conclusions

The extreme solubilization alkaline pH obtained a higher protein recovery yield, and the pH 12.0, 5.5 treatment, pH 5.0 precipitation treatments, and pH 12.0, 6.0 treatment obtained a high recovery yield. The pH 10.5 and pH 11.0 solubilization treatments and pH 12.0, 6.0 treatment showed a good emulsifying capacity with a uniform and stable emulsion distribution and no stratification. Overall, based on the protein recovery yield and the EC results, ISPs using the pH 10.5, 5.0, pH 11.0, 5.0, and pH 12.0, 6.0 treatments are a feasible technique to improve the economic value of CLs as an emulsifier in the food industry. In the future, more in-depth mechanistic studies about the “quantity, structure, and effect” relationship about the ISP CL protein emulsion are warranted to promote the up-scaling of industrial applications.

## Figures and Tables

**Table 1:** Particle size of chicken liver protein recovered using different ISPs.

Solubilization and Precipitation pH	D_3,2_ (μm)	D_4,3_ (μm)
pH 10.5, 5.0	13.056 ± 0.20 ^f^	39.954 ± 0.52 ^d^
pH 10.5, 5.5	10.635 ± 0.04 ^h^	23.539 ± 0.07 ^ef^
pH 10.5, 6.0	9.197 ± 0.50 ^i^	14.576 ± 0.14 ^h^
pH 11.0, 5.0	11.008 ± 0.48 ^gh^	25.425 ± 0.71 ^e^
pH 11.0, 5.5	11.358 ± 0.02 ^g^	19.172 ± 0.04 ^g^
pH 11.0, 6.0	14.525 ± 0.05 ^e^	22.589 ± 0.07 ^f^
pH 11.5, 5.0	15.153 ± 0.56 ^d^	45.534 ± 1.88 ^c^
pH 11.5, 5.5	18.376 ± 0.12 ^b^	44.563 ± 0.92 ^c^
pH 11.5, 6.0	17.794 ± 0.14 ^c^	39.743 ± 0.99 ^d^
pH 12.0, 5.0	14.075 ± 0.42 ^e^	39.858 ± 1.22 ^d^
pH 12.0, 5.5	19.849 ± 0.12 ^a^	53.654 ± 0.43 ^a^
pH 12.0, 6.0	18.499 ± 0.22 ^b^	49.069 ± 3.45 ^b^

Note: Values are the means ± SD (n = 5). Different superscripts (a–i) in the same column indicate significant differences among the different recovered ISPs (*p* < 0.05).

## Data Availability

The data presented in this study are available on request from the corresponding author.
